# The Presentation, Diagnosis, and Management of Autosomal Dominant Common Variable Immunodeficiency Type XII with NFKB1 Mutation and Autoimmune Neutropenia Treated with Allogenic Stem Cell Transplantation

**DOI:** 10.3390/hematolrep17050049

**Published:** 2025-09-22

**Authors:** Matthew Gold, Chandini Kannan, Ashley Schofield, Alane Rogers, Charles J. Weeks, Sruthi Dontu, Joseph Suchomski, Nabil Ghani, Shawn Doss, Jacob Boccucci, Mei Zheng, Amany Keruakous

**Affiliations:** 1 Department of Internal Medicine, Medical College of Georgia, Augusta University, Augusta, GA 30912, USA; mgold@augusta.edu (M.G.); ckannan@augusta.edu (C.K.); jsuchomski@augusta.edu (J.S.); shdoss@augusta.edu (S.D.); jboccucci@augusta.edu (J.B.); 2 Department of Hematology and Oncology, Georgia Cancer Center, Augusta University, Augusta, GA 30912, USA; ashschofield@augusta.edu (A.S.); nghani@augusta.edu (N.G.); mezheng@augusta.edu (M.Z.); 3 Medical College of Georgia, Augusta University, Augusta, GA 30912, USA; alarogers@augusta.edu (A.R.); chweeks@augusta.edu (C.J.W.); sdontu@augusta.edu (S.D.)

**Keywords:** common variable immune deficiency, allogeneic transplant, hematopoietic stem cell, NF-kappa-B protein, Febrile neutropenia

## Abstract

**Background and Clinical Significance:** Common Variable Immunodeficiency (CVID) is a prevalent manifestation of primary immunodeficiency disorder. The current mainstay of treatment is immunoglobulin replacement therapy; however, in patients with severe complications or refractory disease, hematopoietic stem cell transplant (HSCT) is indicated. Despite this, there has been little research regarding HSCT as a treatment for CVID, with few case reports demonstrating clinical benefit. **Case presentation:** We present a unique case of common variable immunodeficiency Type XII (CVID12) with rare NFKB mutation and its management. A 20-year-old female with autoimmune alopecia, eczema, and a congenital atrophic right kidney presented to the emergency department with a three-month history of intermittent fever, malaise, lymphadenopathy, mouth sores, diarrhea, and odynophagia, accompanied by a 5 lb. unintentional weight loss and night sweats. Previously, she received multiple steroid prescriptions for these symptoms, providing only temporary relief with each course. Lab findings revealed severe neutropenia and imaging demonstrated hepatosplenomegaly and lymphadenopathy. Flow cytometry revealed a slightly atypical CD8-positive T-cell population and bone marrow biopsy revealed variable cellular marrow with trilineage hematopoiesis. Genetic testing confirmed the diagnosis of Autosomal Dominant Common Variable Immunodeficiency Type XII with an NFKB1 mutation. Pre-transplant treatments included monthly IVIG, weekly rituximab, and daily filgrastim, all of which failed to improve her autoimmune neutropenia and hypogammaglobulinemia and failed to reduce her symptomatic burden. Given the patient’s young age and refractory autoimmune neutropenia, it was decided to manage them definitively with hematopoietic stem cell transplantation (HSCT). She ultimately underwent allogenic stem cell transplantation (haploidentical, donor was the mother) with 3.96 × 10^8^/kg TNC without immediate post-transplant complications. **Conclusions:** This article demonstrates a rare case of NFKB1-positive CVID that was successfully treated with HSCT and highlights the importance of considering transplant therapy in younger patients with clinically significant, refractory autoimmune cytopenia.

## 1. Background

Common variable immunodeficiency (CVID) is the most common symptomatic manifestation of primary immunodeficiency. The prevalence of CVID in patients diagnosed with a primary immunodeficiency in the United States was found to be 15.8% compared to 11.3% internationally [1]. It has a higher prevalence in North America and Europe, compared to other regions. However, this could be due to outside factors such as availability of diagnostic methods and disease registries used to track global cases.

CVID presents with a highly variable clinical picture, but the pillars of diagnosis are hypogammaglobulinemia and recurrent infection. The European Society of Immunodeficiency (ESID) outlined diagnostic criteria for CVID, which include at least one of the following: increased susceptibility to infection, autoimmune manifestations, granulomatous disease, unexplained polyclonal lymphoproliferation, or affected family members with antibody deficiency. In addition, patients must be older than the age of four, have a marked decrease in IgG and IgA, with secondary causes of hypogammaglobulinemia having been excluded, poor antibody response to vaccines, and no evidence of profound T cell deficiency [2]. It is important to highlight other potential causes of neutropenia that must be excluded in order to definitively diagnose CVID. For instance, drug-induced neutropenia is a frequently documented cause of neutropenia. These adverse effects are typically acute and severe, often presenting within weeks to months of exposure. Viral infections, such as Epstein–Barr Virus (EBV), Cytomegalovirus (CMV), Human Immunodeficiency Virus (HIV), and hepatitis are important causes of transient or persistent neutropenia, particularly in mononucleosis-like syndromes. Autoimmune neutropenia may also be considered secondary to Systemic Lupus Erythematosus (SLE) or Rheumatoid Arthritis (RA). These systemic responses are characterized by neutrophil-specific autoantibodies and peripheral destruction. Nutritional deficiencies and severe protein-calorie malnutrition can also impair granulopoiesis, resulting in neutropenia. Additionally, in the context of chronic liver disease or hematologic disorders, hypersplenism leads to sequestration and the subsequent destruction of neutrophils. Finally, the following pathologies should not be missed in the workup of neutropenia: bone marrow infiltration by malignancy, bone marrow failure syndromes, Myelodysplastic Syndrome (MDS), and Hemophagocytic Lymphohistiocytosis (HLH).

Common variable immunodeficiency-12 (CVID12) is an autosomal dominant subtype of CVID, resulting from genetic mutations of NFKB1 [3]. It presents with autoimmunity, characterized by multisystem involvement, the development of cytopenia, generalized inflammation, and lymphoproliferation. The clinical phenotype was first described in members of a large family, with multiple generations of suspected cases [4]. Severity of disease was highly variable within the family, ranging from overt CVID with hypogammaglobulinemia to milder cases of dysimmunoglobulinemia. Common clinical presentations within this family included recurrent infections and autoimmune disorders like immune thrombocytopenia purpura and autoimmune hemolytic anemia. Through pedigree analysis, researchers determined that the genetic variation in this family was most likely inherited in a pattern of autosomal dominance. Follow-up genomic sequencing studies of this family revealed that the mutation was most likely located on chromosome 4q [5].

In a 2018 study, researchers sequenced the genomes of 846 unrelated patients in Europe with primary immunodeficiency including CVID, which accounted for most of the cohort. They determined that the most common monogenic cause of CVID12 was heterozygous loss of function mutations in NFKB1 and identified 16 distinct genetic variations involving NFKB1 with a pattern of incomplete penetrance. Like the family previously studied, members of this cohort were clinically characterized by hypogammaglobulinemia, splenomegaly, lymphadenopathy, and autoimmune disease. Other genes implicated in CVID may present polygenically or be disease-modifying rather than disease-causing, such as mutations of TACI [6].

Through continued research, an increasing number of variants involving NFKB1 have been identified in patients with CVID12. In a recent study, CVID12 phenotypes associated with various NFKB1 mutations were analyzed [7]. We present a rare case of common variable immunodeficiency type 12 with a NFKB1 mutation not yet described in the literature, c.380del (p. Thr127Metfs*19), and a VUS in AP3D1 (Varsome = LB), associated with refractory autoimmune neutropenia, which was managed successfully with allogenic stem cell transplantation. The patient had a complicated course leading up to the decision to pursue stem cell transplantation. In this manuscript, we will articulate the presentation, diagnosis, and management of this disease with literature review.

## 2. Case Presentation

The patient is a 20-year-old female with pertinent medical history of autoimmune alopecia, eczema, congenital atrophic right kidney, and major depressive disorder, who presented to the emergency department with a three-month history of intermittent periods of relapsing remitting fevers, malaise, pharyngitis, cervical lymphadenopathy, mouth sores, diarrhea, and odynophagia. At this time, she also reported five-pound unintentional weight loss as well as intermittent night sweats. The patient was previously treated by her primary care provider for multiple issues, including several prescriptions of steroids which only resulted in temporary improvement in symptoms. The patient’s family history is significant for renal aplasia and thyroid dysgenesis in her mother. Her father is healthy. No family history of any NFKB1 mutations or other pathogenic genetic mutations is present. Socially, the patient lived in a trailer, where she was exposed to black mold; however, she is without a history of smoking, recreational drug, or alcohol use. She has no known drug allergies. There is no history of any known hospitalizations or childhood illnesses in the patient.

Vital signs at the time show a maximum temperature (T-max) of 38.8 °C, heart rate of 64, respiratory rate of 20, blood pressure of 122/82, and oxygen saturation of 100% in room air. Physical examination was notable for a pale-appearing female with bilateral non-mobile and tender lymphadenopathy below inguinal ligaments, bilaterally enlarged fixed and nontender submandibular lymph nodes, oropharyngeal erythema, and mild erythematous rash of right axilla. Pertinent laboratory findings at the time revealed white blood cell count of 2.23 × 10^9^/L (lymphocyte count of 4 × 10^9^/L, neutrophils of 0.03 × 10^9^/L, monocytes 0.76 × 10^9^/L, eosinophils of 0.03 × 10^9^/L), red blood cell count of 5.45 million/μL, hematocrit of 37.8%, hemoglobin of 13.9 g/dL, mean corpuscular volume of 69.3 fL, platelet count of 151.8 × 10^3^/μL, immunoglobulin (Ig) G 312 mg/dL, IgA 45 mg/dL, IgM 33 mg/dL, and erythrocyte sedimentation rate of 2 mm/h. Computed tomography (CT) of the neck revealed edematous appearance of oropharynx and larynx concerning for laryngitis/pharyngitis, numerous enlarged lymph nodes at level 1B bilaterally, multiple solid enhancing lymph nodes at level 3 bilaterally, and no evidence of retropharyngeal abscess. The CT scan of the chest was unremarkable. The CT of the abdomen and pelvis was notable for hepatosplenomegaly with an atrophic right kidney without visualized right renal artery and with associated compensatory hypertrophy of the functioning left kidney.

An extensive infectious workup was completed to rule out infectious etiologies contributing to this patient’s clinical and laboratory findings. It included screening for Epstein–Barr Virus (EBV), Cytomegalovirus (CMV), Herpes Simplex Virus (HSV), Toxoplasma, and both acute and chronic hepatitis B; all results were negative. Previous titers to hepatitis B surface antibody were noted to be 371.05 mIU/mL, range < 7.99 mIU/mL. Additionally, Human Immunodeficiency Virus (HIV), gastrointestinal viral panel, and respiratory viral panel were all negative. Urine, blood, and fungal cultures were obtained and resulted negative. Group A strep throat culture was also negative. Stool ova and parasite, QuantiFERON, Bartonella, Parvovirus B19 came back negative as well. Additionally, rheumatologic work-up of ANA, HLA-B27, HLA 1 Cl antigen was negative.

**Figure 1:**
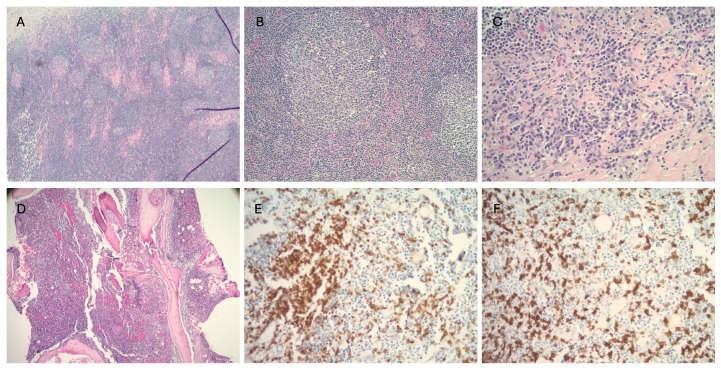
Images (**A**–**C**) are of the Level 2 lymph node. (**A**) (40×) and (**B**) (200×) are hematoxylin and eosin (HE)-stained, showing Lymph node with follicular hyperplasia and T zone expansion. (**C**) (200×) shows HE-stained Lymph node subcapsular reactive plasma cell infiltrate. Images (**D**–**F**) are of the bone marrow biopsy. (**D**) (40×) is HE stained, showing hypercellular marrow with trilineage maturing hematopoiesis with increased interstitial lymphoid infiltrate. (**E**,**F**) (200×) have immunostains CD3 (**E**) and CD8 (**F**) showing increased interstitial CD3 and CD8 positive T cells (TCR gene rearrangement: Negative).

A peripheral blood smear review demonstrated red cell microcytosis and profound neutropenia. Peripheral blood flow cytometry revealed slightly atypical CD8 positive T-cell population, no blast population, and monocytes without aberrancy. Bone marrow biopsy demonstrated variable cellular marrow with trilineage hematopoiesis, including decreased myeloid precursors with maturation arrest and associated eosinophilia and hematocrit. It was negative for any evidence of high-grade dysplasia or increased blasts and revealed a normal karyotype. Next generation sequencing panel performed on the marrow biopsy specimen revealed no aberrations and myelodysplastic FISH panel was negative. Two excisional biopsies of left level 2 nodes demonstrated reactive changes and were negative for neoplasia (Figure 1). Genetic testing confirmed the diagnosis of Autosomal Dominant Common Variable Immunodeficiency Type XII with an NFKB1 mutation—c.380del (p. Thr127Metfs*19), likely to be a de novo mutation of NFKB1, and a VUS in AP3D1—c.2663-2668dup (p. Ala888_Pro880dup).

However, mutations in the AP3D1 gene are associated with Hermansky–Pudlak syndrome type 10 (HPS10), but there is a lack of oculocutaneous albinism, neurologic disorders, bleeding diathesis from platelet granule deficiency, and lack of any pathogenic HPS10 gene variants, since our patient had a variant in AP3D1, not homozygous mutation [8]. Prior to the decision to proceed with transplant, efforts were made to manage the patient with prophylactic antimicrobials, filgrastim, and IVIg. Initially, the patient presented to her primary care where she was treated several times for upper respiratory symptoms, oral ulcers and diarrhea which improved with steroids. Once established with hematology and oncology for her profound neutropenia, she was started on prophylactic acyclovir, fluconazole, and doxycycline. Subsequently, she re-presented with complaints of diarrhea, fevers, and upper airway edema and was found to have *Clostridium difficile*. A colonoscopy at this time showed active colitis with numerous crypt abscesses and active inflammation with decreased plasma cells consistent with CVID and recent infection. She was managed with a temporary course of filgrastim and IVIg resulting in improved counts and clinical symptoms. The patient unfortunately continued to have recurrent presentations with continued complications including recurrent fevers, oral ulcers, and styes. She went through several increases in the frequency of filgrastim and IVIg administration with each presentation for recurrent symptoms and infections. When her symptoms were relatively under control, she was receiving daily filgrastim and weekly IVIg infusions, with an IgG goal of >1000, as her symptoms appeared to have an association with IgG levels falling below this threshold. Due to the quality-of-life impact that this regimen incurred, it was attempted to de-escalate the patient to filgrastim twice weekly and IVIg to every 3 weeks. Unfortunately, her neutrophil and IgG counts dropped again, and she resumed daily filgrastim with the addition of monthly rituximab for immune thrombocytopenia and continued on IVIg. Her counts would recover, but after a week would drop to neutropenic range (Table 1). Despite this, she developed several other neutropenic syndromes, and the decision was made for the patient to pursue allogenic stem cell transplantation for definitive management.

The patient was admitted to the hospital on transplant day -9 to begin conditioning chemotherapy with Anti-thymocyte globulin/Fludarabine/Cyclophosphamide and Total Body Irradiation. The patient was febrile on admission with T-max of 39.3 °C, complaining of mouth pain with left facial edema noted, left common fissure with area of grouped vesicles, tip of tongue with ulceration, and left buccal mucosa with stomatitis. She received cefepime empirically pending the results of the infectious evaluation, such as blood cultures. The lesions of the left common fissure were tested and returned positive for HSV-2. The patient was initiated on a fourteen-day course of valacyclovir with subsequent resolution of lesions. Ultimately, HSV was determined to be the most likely culprit for her fever on admission. Oral vancomycin was started on the day of admission for *Clostridioides difficile* prophylaxis due to her prior infectious history. She was also started on ursodiol for VOD prophylaxis. She was found to be febrile again on the day of transplant (day 0) with T-max of 39.7 °C. At this time, the patient was re-started on cefepime and switched to IV vancomycin. Infectious workup remained unrevealing for new infectious etiology. On day 0, the patient proceeded with haploidentical allogenic stem cell transplantation donated from the patient’s mother with 3.96 × 10^8^/kg TNC, infused without immediate complications. The vancomycin was de-escalated on day 2, followed by cefepime on day 3. The patient remained afebrile for the remainder of admission and her hospital course was uncomplicated.

Graft versus Host disease (GvHD) prophylaxis with cyclophosphamide was started on days +3 and +4 with tacrolimus and mycophenolate beginning on day +5. Tacrolimus levels were monitored and adjusted accordingly. Filgrastim was started on day +5 and continued until neutrophil engraftment. Letermovir for CMV prophylaxis was started on day +9 with the plan for trimethoprim/sulfamethoxazole for PCP prophylaxis on day +21. Neutrophil engraftment occurred on day 19. She was awaiting platelet engraftment at the time of discharge, with patient’s last platelet transfusion on day +13. She was discharged on day +19. At discharge, immunosuppressive therapy consisted of tacrolimus 0.5 mg twice daily and mycophenolate 750mg TIDAC until day 35, in addition to antimicrobial prophylaxis with acyclovir, posaconazole, letermovir, and entecavir.

Post-transplant, the patient has been followed for a year and is doing well. She had one 2-day admission for new-onset diarrhea and facial rash, deemed to be secondary to GvHD. She is currently finishing tapered doses of tacrolimus 0.5 mg daily, ruxolitinib 5 mg twice daily, and budesonide 9 mg daily, along with a tacrolimus cream, with resolution of GI symptoms and marked improvement in skin symptoms. She remains stable with normal blood cell counts.

## 3. Discussion and Conclusions

Common Variable Immunodeficiency subtype XII (CVID12) is a primary immunodeficiency associated with heterozygous NFKB1 mutations. The gene implicated in this complex immunologic dysregulation, NFKB1, is responsible for several important biologic processes, including cell survival and proliferation, inflammation, and adaptive immune responses [9]. While this patient’s specific NFKB1 mutation is yet to be described, other pathologic mutations of NFKB1 have been analyzed. Li et al. 2021 developed a novel assay to determine functional activity in various NFKB1 mutations [10]. They determined that the loss of function mutations of NFKB1 were in the Rel-homology domain (RHD), which is a region responsible for the generation of p50. They supported the concept of p50 haploinsufficiency as an etiology of CVID, based on the results of this assay [9]. Heterozygous NFKB1 mutations causing p50 haploinsufficiency have been demonstrated to play an important role in defining the phenotype of CVID12 [11]. Lorenzini et al. 2020 analyzed the presentation and treatment of heterozygous NFKB1 variants in an effort to curate a more comprehensive clinical description of the spectrum of NFKB1-related phenotypes [7]. The authors explored several treatment strategies for those with autoimmunity and immune dysregulation, including immunoglobulin replacement, steroids, and Rituximab, as well as the cytotoxic T-lymphocyte antigen 4 fusion protein Abatacept [7]. The gene APD31, in which the VUS was found, currently has no well-established disease association; although, there is preliminary evidence suggesting correlation with autosomal dominant Hermansky–Pudlak syndrome, autosomal dominant schizophrenia, and autosomal dominant autism spectrum disorder [8,12,13]. The specific heterozygous VUS found has not been reported in the literature in individuals affected with APD31-related conditions. Moreover, the frequency of this variant in the general population is considered unreliable, given poor data and lack of evidence on its functional significance.

While CVID can typically be managed with the aforementioned therapies, a subset of patients with CVID, such as the patient described in this case report, experience severe complications such as immunologic dysregulation, refractory autoimmunity, or malignancies, necessitating more aggressive treatment approaches. In particular, hematopoietic stem cell transplantation (HSCT) can be considered, as suggested by The American Academy of Allergy, Asthma, and Immunology (AAAAI) guidelines [14]. However, the review of the relevant literature yielded a lack of meaningful investigation into the roles and outcomes of patients treated with hematopoietic stem cell transplantation and more targeted therapeutic options, such as proteasome inhibitors. Other therapies that modulate the NFKB1 pathway include mTOR inhibitors, JAK inhibitors, IL-12/23 inhibitors, BAFF, or CTLA-4 therapies. Very few case reports have demonstrated the benefit of HSCT in CVID patients, predominantly highlighting the high mortality associated with the procedure. Specifically, one multicenter retrospective study on HSCT in cases of complicated CVID found an overall survival of 48% with major causes of death as treatment-refractory GvHD, poor immune reconstitution, and infectious complications.

In this study, the most common indications for HSCT included the development of lymphoma, severe infections refractory to treatment, and immune system dysregulation, such as granulomatous organ involvement or severe cytopenia, as in this patient. Conditioning regimens varied by center and included both myeloablative conditioning (MAC) and reduced-intensity conditioning (RIC). A higher mortality rate was associated with MAC. Long-term follow-up of the surviving patients revealed that 92% had resolution of their primary immunodeficiency, with 83% of the survivors having 100% whole blood donor chimerism following HSCT. Genetic information on complex CVID cases treated with HSCT is limited, and the current literature indicating poor outcomes emphasizes the need for further data on patient selection criteria and timing for HSCT in CVID patients [15,16,17,18].

HSCT was found to be successful in this patient with a rare genetic variant of CVID12, characterized by refractory autoimmune neutropenia. This highlights the need for further research and exploration of hematopoietic stem cell transplantation and NFKB1 pathway-targeted therapies in the future. Additionally, it would be beneficial to continue following this patient to monitor for long-term complications as this could help define how careful patient selection of specific NFKB1-related phenotypes and optimization of transplantation protocols are crucial for improving outcomes. Overall, this article demonstrates a rare case of NFKB1-positive CVID that was successfully treated with HSCT and highlights the importance of considering transplant therapy in younger patients with clinically significant, refractory autoimmune cytopenia.

## Figures and Tables

**Table 1:** Absolute neutrophil trend on four different occasions in the patient at initial diagnosis before treatment, treatment with Filgastrim monotherapy, Filgastrim and rituximab combination, pre-allogenic transplant, post-transplant, and 1 year follow-up.

Absolute Neutrophil Count (ANC) thous/mm^3^					
Initial Diagnosis	Filgrastim	Filgrastim + Rituximab	Pre-Transplant	Immediate Post-Transplant	Approaching 1 Year Follow-Up
0.1	0.6	3.4	0	2.9	6
0	1.4	3	0	2	5.4
0	1.1	1.6	0	1.4	6.5
0.3	0.1	1.4	0	1.5	3.7

## Data Availability

The original contributions presented in this study are included in the article. Further inquiries can be directed at the corresponding author.
